# Potential for high yield with increased seedling density and decreased N fertilizer application under seedling-throwing rice cultivation

**DOI:** 10.1038/s41598-018-36978-w

**Published:** 2019-01-24

**Authors:** Yang Liu, Chao Li, Baohua Fang, Yong Fang, Kailin Chen, Yuzhu Zhang, Haiqing Zhang

**Affiliations:** 1 Hunan Rice Research Institute, Hunan Academy of Agricultural Sciences, Changsha, 410125 China; 2 Southern Regional Collaborative Innovation Center for Grain and Oil Crops (CICGO), Hunan Agricultural University, Changsha, 410128 China; 3 Hunan Hybrid Rice Research Center, Hunan Academy of Agricultural Sciences, Changsha, 410125 China; 4 Hunan Soil and Fertilizer Research Institute, Hunan Academy of Agricultural Sciences, Changsha, 410125 China; 5 Institute of Agricultural Biotechnology, Hunan Academy of Agricultural Sciences, Changsha, 410125 China

## Abstract

Fertilizer application for rice production has increased significantly in southern China to raise yields, but has led to problems with lodging, quality decline and environmental pollution. Therefore, research on fertilizer-saving cultivation technologies for high-yielding rice is necessary. A two-factor experiment was conducted to evaluate the effects of seedling-addition treatment (SAT) and nitrogen-saving treatment (NST) on yield formation and nitrogen absorption of individual plants and plant groups under the seedling-throwing cultivation system. Numbers of spikelets per panicle and filled grains per panicle of individual plants declined under decreased nitrogen application, but was compensated by substantially increased effective panicles number and total number of glumous flowers under SAT. Under the optimal SAT–NST treatments of 18% less N fertilizer and 32% additional seedlings, yield increased 1.79% and 4.29% compared with that of conventional practice (CK) in 2015 and 2016, respectively. The mature-stage individual-plant biomass under SAT–NST treatments decreased by 27.46% and 20.49% compared with CK in 2015 and 2016, but plant-group biomass did not differ significantly (all >16 t ha^−1^). Under SAT–NST treatments, effective number of panicles was positively correlated with maximum number of seedlings (*r* = 0.643) and N absorption amount in the tillering stage (*r* = 0.901).

## Introduction

Rice (*Oryza sativa* L.) is one of the most important grain crops worldwide, especially in Asia^1^. Over the next three decades, population growth and decreasing arable land area will pose a severe challenge to rice production in China, which is projected to use 68 million tons of fertilizer by 2030^2^. To mitigate these problems, China’s Ministry of Agriculture proposed guidelines to reduce the use of chemical fertilizers and pesticides to develop sustainable agricultural production. Nitrogen (N) is essential to achieve high rice yields. For example, Zhu *et al*.^3^ reported that the optimum quantity of N fertilizer to increase grain yield was 225–300 kg ha^−1^. Fu *et al*.^4^ suggested that the N application rate for super rice should be increased by 12–14% to obtain high yield. These studies show that increases in rice yield rely heavily on excessive application of N fertilizer.

However, excessive N application significantly reduces the quality of rice, and leads to higher N loss and soil acidification^5–7^. Plant density is one of the main factors that influence yield in rice, together with N availability^8,9^. Zhu *et al*.^10^ observed that a moderate planting density combined with reduced basal application of N fertilizer can lead to rice crops with high yield and high N-use efficiency. Martinez *et al*.^11^ observed that at high plant densities, phyllochrons were longer, panicle initiation occurred earlier, and the total number of leaves on the main stem decreased.

Seedling-throwing is an alternative planting method for rice production popular in southern China because it may save on labor input and promote high yields. The seedling-throwing cultivation system also has potential benefits for reduction of fertilizer inputs. However, research into this cultivation practice is limited, especially with regard to its N-saving potential^10,12^. The objectives of the present research were to develop high yield potential in the seedling-throwing rice cultivation system by means of an appropriate increase in seedling density and a concomitant decrease in N fertilizer input, and to explore the mechanisms for high yield and N conservation.

## Results

### Grain yield and yield components

Averaged across nine treatments, the grain yield were 7.84 t ha^−1^ in 2015 and 7.45 t ha^−1^ in 2016 (Table 1). The differences in grain yield among the treatments were significant, although the magnitude of the differences between 2015 and 2016 varied. The grain yield of the CK in 2015 and 2016 was 8.37 t ha^−1^ and 7.69 t ha^−1^, respectively. Under seedling-addition treatment (SAT) (T1 and T2) or nitrogen-saving treatment (NST) (T3 and T4) the grain yield was decreased compared with that of the CK: T1 and T2 yields decreased by 1.30–6.69%, and T3 and T4 yields decreased by 4.42–12.78%. The grain yield of combined SAT–NST treatments (T5, T7 and T8) decreased by 1.30–13.74%. But the grain yield of T6 increased by 1.79% and 4.29% compared with that of the CK in 2015 and 2016, respectively, but only the increase in 2016 was significant.

**Table 1:** Grain yield of early-season rice ‘Xiangwanxian 45’ under nitrogen fertilizer and plant density treatments in 2015 and 2016.

Year		2015			2016		
Treatment		Yield (t·ha^−1^)	Yield increased vs. CK (t·ha^−1^)	Increase ratio (%)	Yield (t·ha^−1^)	Yield increased vs. CK (t·ha^−1^)	Increase ratio (%)
SAT	T1	7.81bc	−0.56	−6.69	7.59b	−0.10	−1.30
	T2	7.85b	−0.52	−6.21	7.40b	−0.29	−3.77
NST	T3	7.84b	−0.53	−6.33	7.35b	−0.34	−4.42
	T4	7.30c	−1.07	−12.78	7.02c	−0.67	−8.71
SAT-NST	T5	8.22ab	−0.15	−1.79	7.59b	−0.10	−1.30
	T6	8.52a	0.15	1.79	8.02a	0.33	4.29
	T7	7.22c	−1.15	−13.74	7.16c	−0.53	−6.89
	T8	7.46c	−0.91	−10.87	7.21bc	−0.48	−6.24
CK		8.37a	—	—	7.69b	—	—

Within the row for each location, means followed by the same letters are not significantly different according to LSD at P = 0.05.

With regard to yield components for plant groups, the total number of spikelets and effective panicle number increased under SAT (T1 and T2) and decreased under NST (T3 and T4). Combined SAT–NST had a significant interaction effect on these two traits. The total number of spikelets in the T6 treatment was the highest among all treatments and exceeded that of the CK by 12.17% in 2015 and 5.82% in 2016. But in the T7 and T8 treatments declined significantly (Tables 2 and 3). The panicle number increased significantly under increased seedling density. Under combined SAT–NST, the panicle number in the T6 treatment was increased by 11.98%, 26.61%, 13.26% and 20.73% compared with that of T5, T7, T8 and CK in 2015 and by 8.30%, 18.22%, 12.91% and 14.76% in 2016, respectively.

**Table 2:** Yield components of early-season rice ‘Xiangwanxian 45’ under nitrogen fertilizer and plant density treatments in 2015.

Treatment		Total Spikelets (10^7^·ha^−1^)	Panicle No. (10^4^·ha^−1^)	Spikelets per panicle	Filled grains per panicle	Spikelet filling percentage (%)	1000-grain weight (g)
SAT	T1	4.36ab	428.7b	101.6c	81.5d	80.2c	23.6ab
	T2	4.62a	460.6a	100.2c	74.4e	74.3d	23.5ab
NST	T3	4.08bc	374.0c	106.4ab	90.8ab	85.3ab	23.6ab
	T4	3.75c	345.1d	108.7a	94.6a	87.0a	23.2b
SAT-NST	T5	4.29ab	415.5bc	103.2bc	84.6c	82.0b	23.7ab
	T6	4.70a	465.3a	101.1c	81.9d	81.0b	23.2b
	T7	3.82c	367.5cd	103.9b	86.1bc	82.9b	23.6ab
	T8	4.17b	410.8b	101.4c	83.3cd	82.1b	23.4ab
CK		4.19b	385.4c	108.7a	92.9a	85.5ab	23.9a
**Analysis of variance**							
seedling (S)		*	**	*	**	*	ns
nitrogen (N)		*	*	ns	ns	ns	*
S × N		*	**	*	*	ns	ns

Within the row for each location, means followed by the same letters are not significantly different according to LSD at P = 0.05.

*Significant at the 0.05 level based on analysis of variance.

**Significant at the 0.01 level based on analysis of variance.

Ns denotes non-significance based on analysis of variance.

**Table 3:** Yield components of early-season rice ‘Xiangwanxian 45’ under nitrogen fertilizer and plant density treatments in 2016.

Treatment		Total Spikelets (10^7^·ha^−1^)	Panicle No. (10^4^·ha^−1^)	Spikelets per panicle	Filled grains per panicle	Spikelet filling percentage (%)	1000-grain weight (g)
SAT	T1	38.82b	358.8b	108.2b	89.7cd	82.9b	23.6ab
	T2	39.73ab	381.3a	104.2c	84.9e	81.5c	23.3b
NST	T3	36.36c	323.8d	112.3a	94.5b	84.1b	23.3b
	T4	35.69c	314.8e	113.4a	98.3a	86.7a	23.9a
SAT-NST	T5	38.58b	355.3b	108.6b	90.9c	83.6b	23.5ab
	T6	40.55a	384.8a	105.4c	88.7d	84.2b	23.1b
	T7	36.84c	325.5d	113.2a	93.6b	82.7b	23.5ab
	T8	36.39c	340.8bc	106.8bc	90.5c	84.7b	23.4b
CK		38.32b	335.3cd	114.3a	96.6ab	84.5b	23.5ab
Analysis of variance							
seedling (S)		ns	**	*	*	*	ns
nitrogen (N)		**	*	ns	ns	*	ns
S × N		*	**	*	*	ns	ns

Within the row for each location, means followed by the same letters are not significantly different according to LSD at P = 0.05.

*Significant at the 0.05 level based on analysis of variance.

**Significant at the 0.01 level based on analysis of variance.

Ns denotes non-significance based on analysis of variance.

With regard to yield components for individual plants, the number of spikelets per panicle and number of filled grains per panicle decreased significantly under SAT (T1 and T2) compared with those of the CK, but differences between NST (T3 and T4) and CK were less distinct. Combined SAT–NST had a significant interaction effect on these two yield components. Under SAT–NST, the number of spikelets per panicle and number of filled grains per panicle were lowest in the T6 treatment. The number of spikelets per panicle was 101.1 in 2015 and 105.4 in 2016, which was 7.00% and 7.78% less than that of the CK, respectively. The number of filled grains per panicle was 81.9 in 2015 and 88.7 in 2016, which was 11.84% and 8.18% less than that of the CK, respectively. SAT significantly affected the percentage of filled spikelets in 2015 and 2016, whereas NST significantly affected 1000-grain weight in 2015 and percentage of filled spikelets in 2016. However, no interaction effect on percentage of filled spikelets and 1000-grain weight under SAT–NST was observed.

**Figure 1:**
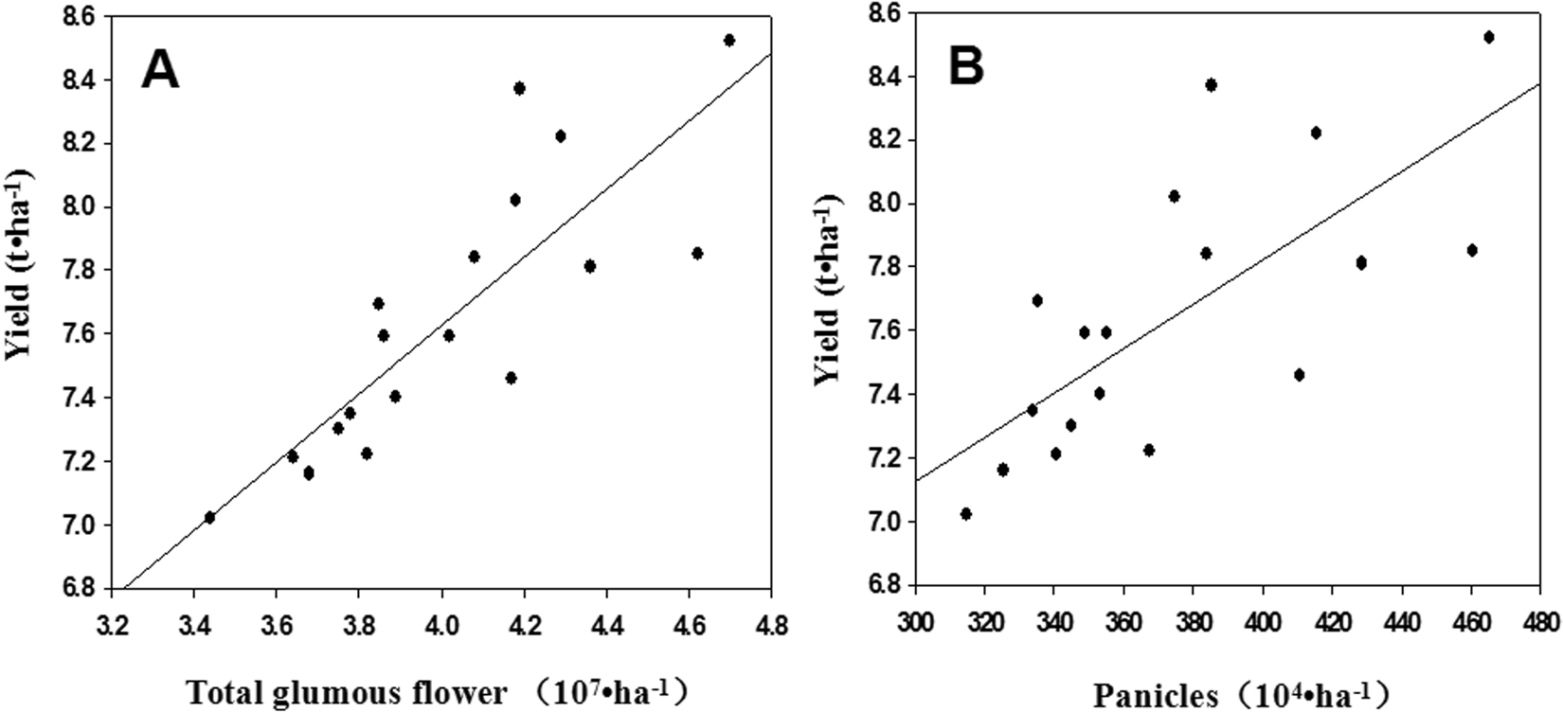
Correlations between total glumous flower, effective panicle number and grain yield. (**A**) Correlations between total glumous flower and grain yield. (**B**) Correlations between effective panicle number and grain yield.

**Figure 2:**
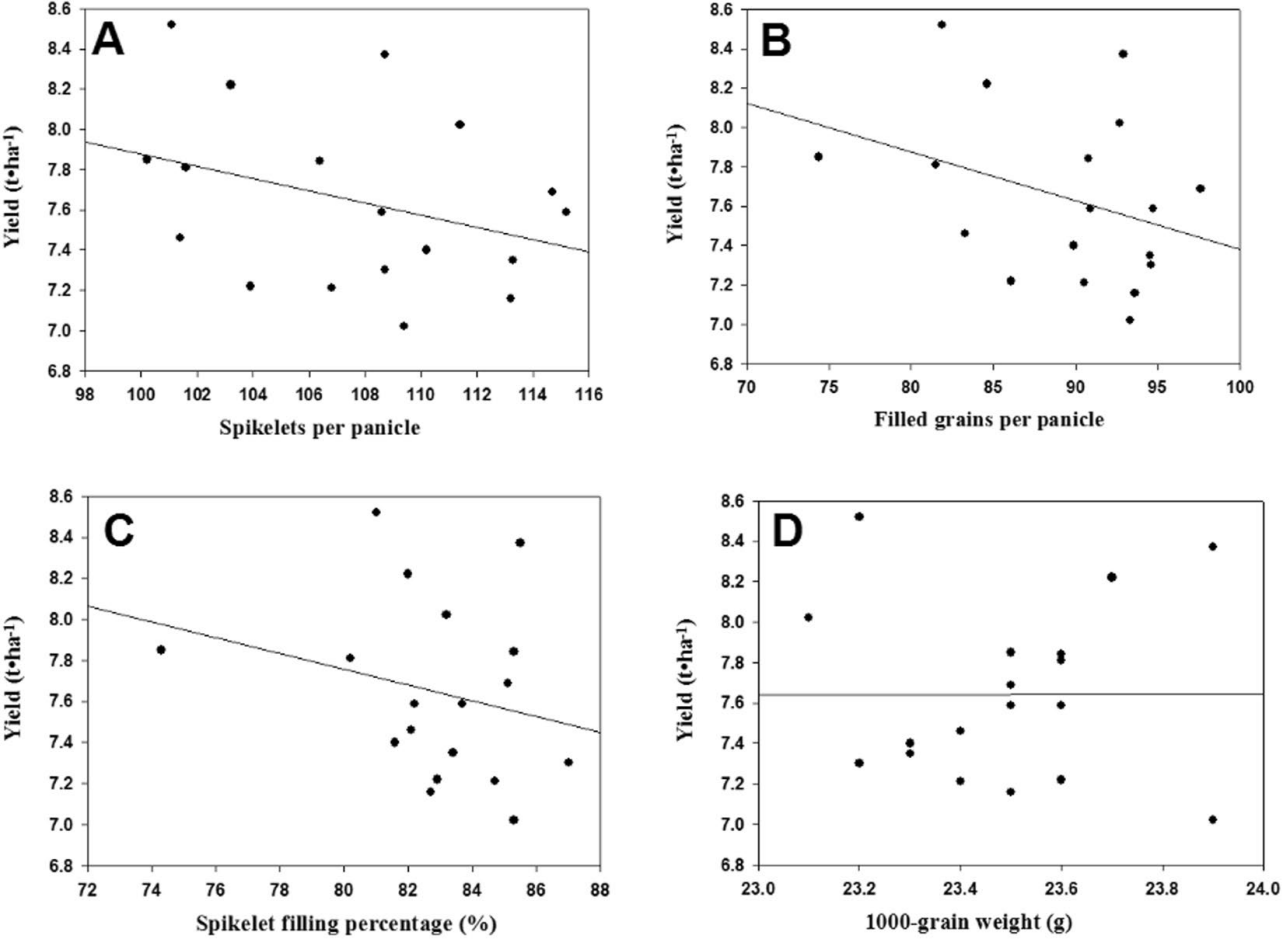
Correlations between spikelets per panicle, filled grains per panicle, spikelet filling percentage 1000-grain weight and grain yield. (**A**) Correlations between spikelets per panicle and grain yield. (**B**) Correlations between filled grains per panicle and grain yield. (**C**) Correlations between spikelet filling percentage and grain yield. (**D**) Correlations between 1000-grain weight and grain yield.

Under combined SAT–NST treatment, the number of spikelets per panicle and number of filled grains per panicle decreased, but the total number of glumous flowers was increased mainly because the number of effective panicles greatly increased. The yield of early-season rice was positively correlated with the total number of glumous flowers (*r* = 0.837) and effective panicle number (*r* = 0.724) (Fig. 1A,B). On the other hand, the yield of early-season rice was negatively correlated with the number of spikelets per panicle, number of filled grains per panicle, percentage of filled spikelets and 1000-grain weight (Fig. 2A–D).

### Individual and group biomass production

Significant differences in both individual biomass production (IBP) and group biomass production (GBP) under SAT and NST were observed. The interaction between SAT and NST on these traits was significant. The IBP declined under increased seedling density. Under conventional-practice N treatments, the IBP of T1 was higher 1.46 and 3.04 g plant^−1^ than that of T2 in 2015 and 2016. Under 18% and 36% less N treatments, the IBP of T5 and T7 were higher than that of T6 and T8 respectively. The IBP of combined SAT–NST (T5, T6, T7 and T8) was significantly lower than that of the CK by 16.80–44.24% in 2015 and by 14.66–38.51% in 2016.

The differences in GBP among all treatments differed from those observed for IBP. The GBP was elevated with increased seedling density (Table 4). Under conventional N treatments, the GBP of T2 was higher 1.38 and 0.87 t ha^−1^ than that of T1 in 2015 and 2016. Under 18% and 36% less N treatments, the GBP of T6 and T8 were higher than T5 and T7 respectively. The difference in GBP between combined SAT–NST and the CK was more weakly significant than that observed for IBP, whereas the GBP of T5 and T6 were similar to or surpassed that of the CK.

**Table 4:** Dry matter weight of early-season rice ‘Xiangwanxian 45’ at mature stages under nitrogen fertilizer and plant density treatments in 2015 and 2016.

Treatment		2015		2016	
		IBP (g/plant)	GBP (t·ha^−1^)	IBP (g/plant)	GBP (t·ha^−1^)
SAT	T1	42.31d	13.33b	49.77b	14.01c
	T2	40.85d	14.71b	46.73bc	14.88bc
NST	T3	57.11b	15.42ab	55.93a	15.10b
	T4	42.58d	12.50c	43.15c	12.65d
SAT-NST	T5	51.47c	16.05a	50.92b	16.04ab
	T6	44.87d	16.21a	47.44bc	17.08a
	T7	35.55e	11.97d	39.46d	12.43d
	T8	34.49e	12.41cd	36.69d	13.21cd
CK		61.86a	16.70a	59.67a	16.38a

Within the row for each location, means followed by the same letters are not significantly different according to LSD at P = 0.05.

## Discussion

### High-yield mechanism

As reported in previous studies^13,14^, N fertilization and seedling density had no effect on aboveground biomass production. Other authors have concluded that N fertilization is needed to achieve maximum biomass production^15–17^. In the present study, under the combined SAT–NST treatments the biomass of individual plants declined at the mature stage, but an increase in plant group biomass was achieved through an increase in seedling density, even though N consumption was reduced.

Suitable N application rates and plant spacing in rice fields may improve the growing environment of plants and produce higher grain yield^9^. Under N deficiency, yield components are reduced, especially panicle number per plant and per unit area^18^. By analyzing yield components we observed that reduction in the application quantity of N fertilizer changed yield components of individual plants (number of spikelets per panicle and number of filled grains per panicle) insignificantly, whereas a group index (panicle number) was reduced drastically. It is widely accepted that a high yield potential of rice requires a large number of effective panicles^8,19^. However, the effective panicle number can be increased significantly by means of an appropriate increase in seedling density.

### N-saving mechanism

Given that uniformity of N absorption at different development stages changes rapidly under different cultivation systems, biomass alone or tissue N concentration alone are not reliable indicators of plant N requirements^20^ and may explain only slightly more than 60% of variability in total N accumulation in rice^12^. In the present research, both biomass and tissue N concentration were measured to provide more reliable estimates of plant N absorption regularity under the seedling-throwing cultivation system. The N absorption amount (NAA) in the T1 to T8 treatments was lower than that of the CK by 6.7–34.8% over the entire growth period (Table 5). In addition, NAA was elevated with increase in seedling density and decrease in fertilizer N application rate. The highest N demand by rice plants is during the tillering stage, but a high application rate of N fertilizer as a basal dressing might cause considerable N loss because of the lower demand and uptake capacity of rice seedlings during early growth stages^21^. Under the optimal SAT–NST treatment (T6), 18% less N fertilizer was applied but a higher quantity of N was absorbed during sowing to tillering stage (Table 5) because of the increased seedling density, thus the treatment not only promoted seedling growth but also avoided excessive loss of fertilizer. Kamiji^22^ and Sui^23^ suggested that topdressing with N fertilizer at the panicle initiation stage is an effective and necessary practice to enhance rice spikelet production to achieve high grain yield. Under the SAT–NST treatments, the highest NAA attained was 64.0 kg ha^−1^ during the mature stage, which represents a N absorption proportion (NAP) of more than 40% in the entire growth period.

**Table 5:** Nitrogen absorption regularity of early-season rice ‘Xiangwanxian 45’ at different growth stages under nitrogen fertilizer and plant density treatments in 2016.

Treatment and stage		NAA (kg·ha^−1^)				NAP (%)		
		SO–AT	AT–HD	HD–MA	Whole growth period	SO–AT	AT–HD	HD–MA
SAT	T1	41.3	65.4	40.8	147.5	28.00	44.34	27.66
	T2	47.0	69.7	41.4	158.1	29.73	44.09	26.19
NST	T3	31.6	51.3	47.1	130.0	24.31	39.46	36.23
	T4	26.3	41.7	42.0	110.0	23.91	37.91	38.18
SAT-NST	T5	38.0	55.6	62.2	155.8	24.39	35.69	39.92
	T6	51.7	43.4	64.0	159.1	32.50	27.28	40.23
	T7	48.7	21.8	40.0	110.5	44.07	19.73	36.20
	T8	49.8	19.6	41.6	111.0	44.86	17.66	37.48
CK		41.2	74.8	54.6	170.6	24.15	43.85	32.00

SO–AT, sowing to active tillering; AT–HD, active tillering to full heading; HD–MA, full heading to maturity.

The seedling establishment phase is important for the productivity and profitability of rice^24^. The development of a tiller primordium is influenced primarily by the amount of N supplied to the developing tiller. The total N content of the whole straw, leaves or stem may be a good indicator of the tillering activity of the plant because there are strong correlations between the N content of the leaves and that of the stem, and between the total- and soluble-N content in the culm^25^. In the present study we observed a correlation between maximum number of seedlings and group NAA over the entire growth period under SAT–NST treatments (Table 6). The NAA in the periods from sowing to active tillering, and from active tillering to full heading was positively correlated with the number of effective panicles (*r* = 0.643) and the percentage of available tillers (*r* = 0.619), respectively. Furthermore, the maximum number of seedlings was positively correlated with effective number of panicles (*r* = 0.901) and percentage of available tillers (*r* = −0.839), which is consistent with previous research^10,18^.

**Table 6:** Pearson correlation coefficients between plant traits and nitrogen (N) absorption.

items		Absorptive amount of N			Maximum number of seedlings	Percentage of available tiller	Effective panicles
		SO–AT	AT–HD	HD–MA			
absorptive amount of N	SO–AT	—	*ns*	*ns*	*ns*	*ns*	*
	AT–HD	*r* = −0.307	—	*ns*	*ns*	*	*ns*
	HD–MA	*r* = −0.144	*r* = 0.315	—	*ns*	*ns*	*ns*
the maximum number of seedlings		*r* = 0.483	*r* = 0.349	*r* = 0.027	—	**	**
percentage of available tiller		*r* = −0.321	*r* = −0.618	*r* = 0.228	*r* = −0.839	—	*
effective panicles		*r* = 0.643	*r* = 0.229	*r* = 0.251	*r* = 0.901	*r* = −0.642	—

SO–AT, from sowing to active tillering; AT–HD, from active tillering to full heading; HD–MA, from full heading to maturity.

*Significant at the 0.05 level based on analysis of variance.

**Significant at the 0.01 level based on analysis of variance.

Ns denotes non-significance based on analysis of variance

## Conclusion

This study used the rice seedling-throwing cultivation system practiced in southern China to investigate the potential for attaining high yield under decreased N fertilizer input and increased seedling density with an early-season rice cultivar. By increasing the seedling density by 32% (to 36.0 × 10^4^ seedlings ha^−1^), N application can be reduced by 18% (to 135 kg N ha^−1^) compared with the conventional seedling-throwing practice, but grain yield was not significantly affected. Under combined SAT–NST treatments, the biomass of an individual plant was less than that attained under the conventional practice, but the group biomass was more than 16.0 t ha^−1^, which is identical to that achieved under the conventional cultivation system. The number of spikelets per panicle, number of filled grains per panicle and percentage of filled spikelets decreased when N fertilizer input was reduced, but the number of effective panicles and total number of glumous flowers increased considerably as a result of the increased seedling density, which compensated for the decreased yield of individual plants. On the other hand, under SAT–NST treatments, an increased quantity of N is applied in the tillering and grain-filling stages, which favours a high yield by promoting tiller establishment and development into effective panicles. We conclude that, on the basis of the conventional seedling-throwing cultivation system, the N application rate can be decreased by about 18% to achieve high yield through an appropriate increase in seedling density. The present results provide a theoretical basis for seedling-throwing rice production and are applicable in southern China for the development of sustainable agriculture.

## Materials and Methods

### Field experiments

Field experiments were conducted in Yiyang County (24°03′52″N, 112°03′27″E, 70 m elevation), Hunan Province, China. ‘Xiangwanxian 45’, an inbred *indica* rice cultivar (‘Zhouyou903’ × ‘Zhefu504’), was used as the experimental material. Treatments were arranged in a split-plot design with N treatments as the main plots and seedling density as subplots. The experiment was replicated three times, and the subplot size was 20 m^2^. The three N fertilizer treatments were (i) conventional-practice N application (165 kg N ha^−1^), (ii) 18% less N (135 kg N ha^−1^) and (iii) 36% less N (105 kg N ha^−1^). The three seedling density treatments were (i) conventional-practice density (27.0 × 10^4^ plants ha^−1^), (ii) 16% additional seedlings (31.3 × 10^4^ plants ha^−1^) and (iii) 32% additional seedlings (35.6 × 10^4^ plants ha^−1^). Altogether, there were nine combinations of treatments, which were labeled T1 to T8 and CK (representing the conventional cultivation practice as the control); details of the treatments are listed in Table 7.

**Table 7:** Details of the experimental design and treatments.

Treatment		N dosage (kg·ha^−1^)	Seedlings (10^4^ plant ha^−1^)	Compared with CK	
				Adding seedling	Saving N
seedling-addition treatment (SAT)	T1	165	31.3	16%	0%
	T2	165	35.6	32%	0%
nitrogen-saving treatment (NST)	T3	135	27.0	0%	18%
	T4	105	27.0	0%	36%
Both add seedling and save nitrogen (SAT-NST)	T5	135	31.3	16%	18%
	T6	135	35.6	32%	18%
	T7	105	31.3	16%	36%
	T8	105	35.6	32%	36%
Conventional cultivation	CK	165	27.0	—	—

Pre-germinated seeds were planted in a seedling bowl-tray on 24 March 2015 and 28 March 2016. After leveling of the field, 5–6 seedlings per plant site were thrown into the puddle field on 20 April 2015 and 25 April 2016. When transplanting, the three seedling density treatments (27.0 × 10^4^, 31.3 × 10^4^ and 35.6 × 10^4^ plants ha^−1^) were applied as densities of 15 × 24 cm, 16 × 20 cm and 16 × 20 cm, respectively. Approximately 60% of the N fertilizer was incorporated as basal fertilizer applied one day before seedling-throwing. The remaining N fertilizer was broadcast as urea at the tillering and booting stages, with 20% applied at each application. A total of 150 kg ha^−1^ of potassium was applied to each plot in the form of potassium chloride, with 50% applied as a basal dressing and 50% as a topdressing at panicle initiation. A total of 75 kg ha^−1^ of phosphorus was applied to each plot in the form of superphosphate, with 100% applied as a basal dressing. Except for mid-season drainage, the field was continuously flooded with 3–5 cm water depth until one week before the final harvest. Insects, weeds and diseases were controlled as requiredusing standard practices to avoid yield loss.

### Sampling and measurements

Five plants were sampled in each subplot at active tillering, booting (the fourth booting stage), full heading (when approximately 80% of the panicles had emerged from the flag leaf sheath) and maturity. Plants along the three border lines were excluded from sampling to avoid border effects. Plant samples were separated into stems, leaves and panicles. The dry weight of each organ was determined after oven-drying at 85 °C to a constant weight. Plants sampled at the maturity stage were hand-threshed after the panicles had been counted. We then measured individual indices: filled spikelets were separated from unfilled spikelets by submerging them in tap water; each subsample of 30 g filled grains and all unfilled spikelets were used to count the numbers of spikelets; the dry weight of straw (including the rachis) and numbers of filled and unfilled spikelets were determined after oven-drying at 70 °C to a constant weight; finally, the percentage of filled spikelets (100 × filled spikelet number/total spikelet number) was calculated. The group indices of grain yield and effective panicle number were determined for the total area of each subplot and the yield adjusted to a moisture content of 0.14 g H_2_O g^−1^ fresh weight. The micro-Kjeldahl method was used to determine the N content of all plant samples.

Biomass production and N uptake were calculated using the following formulas.

“Individual” refers to one plant from among the 5–6 seedlings growing at one plant site. Individual biomass production (IBP) reflects the growth of individual plants and was calculated as:

$${\rm{IBP}}={\rm{stem}}\,{\rm{dry}}\,{\rm{weight}}+{\rm{leaf}}\,{\rm{dry}}\,{\rm{weight}}+{\rm{panicle}}\,{\rm{dry}}\,{\rm{weight}}$$

“Group” includes all plants growing within the same subplot. Group biomass production (GBP) reflects the yield potential within the plot and was calculated as:

$${\rm{GBP}}={\rm{IBP}}\times {\rm{plant}}\,{\rm{number}}\,{\rm{per}}\,{\rm{hectare}}.$$

Nitrogen uptake was calculated using the following formulas:

$$\begin{array}{c}{\rm{N}}\,{\rm{absorptive}}\,{\rm{amount}}\,({\rm{NAA}})={\rm{stem}}\,{\rm{dry}}\,{\rm{weight}}\times {\rm{N}}\,{\rm{content}}\,{\rm{in}}\,{\rm{stem}}+{\rm{leaf}}\,{\rm{dry}}\\ {\rm{weight}}\times {\rm{N}}\,{\rm{content}}\,{\rm{in}}\,{\rm{leaf}}+{\rm{panicle}}\,{\rm{dry}}\,{\rm{weight}}\times {\rm{N}}\,{\rm{content}}\,{\rm{in}}\,{\rm{panicle}}\\ {\rm{N}}\,{\rm{absorption}}\,{\rm{proportion}}\,({\rm{NAP}})={\rm{N}}\,{\rm{uptake}}\,{\rm{in}}\,{\rm{a}}\,{\rm{certain}}\,{\rm{period}}/{\rm{N}}\,{\rm{uptake}}\,{\rm{in}}\,{\rm{the}}\,{\rm{entire}}\\ {\rm{growth}}\,{\rm{period}}\end{array}$$

where a certain period includes three phases: SO–AT, sowing to active tillering; AT–HD, active tillering to full heading; and HD–MA, full heading to maturity.

### Statistical analysis

Analysis of variance (ANOVA) was performed using Duncan’s multiple-range test to compare treatment means and significance was defined at *P* < 0.05. Pearson correlation coefficients were calculated using SPSS 10.0.
